# Geographically Indexed Referral Databases to Address Social Needs in the Emergency Department

**DOI:** 10.5811/westjem.2020.11.49250

**Published:** 2021-03-04

**Authors:** Alexa Curt, Hazar Khidir, Gia Ciccolo, Carlos A. Camargo, Margaret Samuels-Kalow

**Affiliations:** * Massachusetts General Hospital, Department of Emergency Medicine, Boston, Massachusetts; † Harvard Affiliated Emergency Medicine Residency, Boston, Massachusetts

## Abstract

**Introduction:**

Unmet health-related social needs (HRSN) are among the drivers of disparities in morbidity and mortality during public health emergencies such as the novel coronavirus 2019 (Covid-19) pandemic. Although emergency departments (ED) see a high volume of patients with HRSN, ED providers have limited time to complete detailed assessments of patients’ HRSN and are not always able to provide up-to-date and comprehensive information to patients on available community resources. Electronic, geographically indexed resource database systems have the potential to provide an efficient way for emergency physicians to rapidly identify community resources in settings where immediate social work consultation is not accessible.

**Methods:**

We conducted a systematic review of papers examining the use of geographically indexed resource database systems in healthcare to better understand how these services can be used in emergency care. We then conducted simulated, standardized searches using two nationally available databases (211 and Aunt Bertha), applied to a single metropolitan area (Boston).

**Results:**

Our systematic review found that most public health and screening interventions using nationally available databases have focused on chronic care needs. A small subset of publications demonstrated that these databases were mobilized during disasters to successfully aid vulnerable populations during Hurricanes Katrina and Rita. A total of 408 standardized searches were conducted to identify community resources related to four domains of social needs (food, transportation, housing, and utilities). Although 99% of the resources identified by both databases were relevant to the search domains queried, a significant proportion of the resources identified by each database were restricted to a specific demographic (eg, veterans).

**Conclusion:**

Our findings demonstrate that geographically indexed referral databases may be an effective tool to help ED providers connect patients to nearby community resources during public health emergencies. We recommend that EDs select a referral database based on the greatest number of resources that are not demographically restricted.

## INTRODUCTION

Public health emergencies, such as the novel coronavirus 2019 (COVID-19) pandemic, have the potential to disproportionately harm vulnerable populations.^1^–^3^ Socially vulnerable populations face a greater likelihood of mortality during public health emergencies.^4^–^5^ For example, during the 2009 H1N1 influenza pandemic, there were higher hospitalization and mortality rates among people who were categorized as low income and those living in low-income communities.^6^,^7^

Unmet health-related social needs (HRSN) are among the primary drivers of disparities in morbidity and mortality during public health emergencies.^1^,^2^ Limited access to healthy food options often result in higher incidence of heart disease, diabetes, and lung disease, which have been linked to increased risk of mortality from the current COVID-19 pandemic.^8^ Patients with unmet HRSN face unique challenges to following emergency preparedness recommendations, such as self-isolation precautions and social distancing. These challenges include cohabitation with multiple family members or friends, unstable housing, inability to stockpile food, limited access to private transportation, limited social networks, lack of childcare, and reliance on income from jobs with limited benefits that pose additional health risks in the setting of an infectious pandemic.^9^,^10^ Additionally, vulnerable patients may have more barriers to accessing care when they or someone in their household become ill enough to require an evaluation, and, therefore, may present increased risk for community spread. For COVID-19, this is particularly concerning given that household contacts have six times higher odds of infection.^11^,^12^

Emergency department (ED) providers often see a high proportion of patients with HRSN.^13^–^15^ Although ED providers are often interested in addressing HRSN and recognize the high rate of unmet social needs affecting their patients, most report they do not feel adequately prepared to solve these problems.^13^–^15^ ED providers have limited time to complete detailed assessments of patients’ HRSN and are not practically able to provide up-to-date and comprehensive information to patients on resources available in their communities. Resources can cover a variety of needs, including help paying the utility bill, finding food pantries or subsidized groceries, and coordinating transportation to medical appointments. Although some EDs have care coordinators and social work services, availability of services is largely limited to daytime hours and some care coordination services may be restricted to certain payer groups (eg, Medicaid).

Geographically indexed resource databases represent a promising strategy for ED providers to efficiently link patients to accessible community resources. These software databases provide an electronic directory of community resources and have the capacity to facilitate e-referrals to social service agencies. Several resource databases are available, but no standardized comparison of their utility in the ED exists. We conducted a systematic review of papers examining the use of the geographically indexed resource database systems in healthcare to better understand how these services can be used in emergency care. We then conducted simulated, standardized searches using two nationally available databases (211 and Aunt Bertha), applied to a single metropolitan area (Boston). The goal of this work was to provide guidance to ED providers looking to develop a local resource directory for their community, specifically about how those databases have been used in the past and the advantages and disadvantages of standard, commercially available databases.

**Population Health Research Capsule**

What do we already know about this issue?

*Emergency department providers see a high proportion of patients with unmet social needs who are at highest risk of morbidity and mortality during public health emergencies*.

What was the research question?

How can geographically indexed resource referral databases be used to address the health-related social needs of patients in the emergency department setting?

What was the major finding of the study?

*Geographically indexed resource referral databases can accurately identify local community resources to meet the common domains of social needs; however, identified resources are oftentimes restricted to a particular patient demographic group*.

How does this improve population health?

*Geographically indexed referral databases may be an effective tool to help ED providers connect patients to nearby community resources during public health emergencies*.

## METHODS

### Scoping Review

We performed a scoping review of published articles discussing geographically indexed databases (eg, 211 system, NowPow, and Aunt Bertha).^17^–^19^ Articles were found via Google, Google Scholar, Ovid Medline, PubMed, and a modified snowball sampling using the cited sources of the primary articles for relevant studies. Search terms included the following: “United Way,” “press/call/dial 211,” “helpline,” “hotline,” “call center,” “caller,” “telephone,” “information services,” “community services,” “crisis intervention,” “hotline services,” “referral,” “consultation” and “professional referral,” “Aunt Bertha,” “CommunityRx,” “NowPow,” “social determinants of health,” “referrals,” “e-prescribing,” “HealtheRx,” and “community services,” “social and medical care,” and “medical informatics.” Inclusion criteria were articles available in English that discussed the social resource database in any capacity. Three team members reviewed articles for eligibility. After duplicates were removed, 41 sources were identified by web-based and database searches.

### Resource Database Usability

We selected two nationally available social resource databases to demonstrate the functionality of new referral technologies when used from the perspective of the ED setting. We chose 211 because it is freely available throughout the United States. We chose Aunt Bertha because it is now the largest database for social services nationally and the basic search functions are freely available despite being a privately run service.^18^ The 211 system began on July 21, 2000, when the Federal Communications Commission (FCC) ruled in favor of assigning the three digit “211” number to social services.^20^ The goal of 211 is to serve a function analogous to 911, but for community referral needs rather than acute medical emergencies; 211 services are available to about 95% of people in the United States as of 2019.^17^ In 2018 alone, 211 centers assisted with 12.8 million requests for community resources.^17^

Aunt Bertha was started in 2010. It is a public benefit corporation that offers an open access, free search engine for public users as well as for-cost features designed for health organizations. Additional features include a social determinants of health screening tool, a closed-loop referral system that provides updates on the status of referrals that are made on a patient’s behalf, and analytical reports for healthcare organizations to measure use.^18^

We simulated the application of the two databases within the large metropolitan area of Boston. We selected 51 ZIP codes to apply to our search comparison to encompass neighborhoods within or surrounding Boston. Searches were conducted between March 2–November 6, 2019 by trained research assistants. For each ZIP code, we ran standardized searches for community resources that related to four domains of social needs (food, transportation, housing, and utilities) across the 211 and Aunt Bertha databases. Search terms for Aunt Bertha included the following: “emergency food,” “food pantry,” “help pay for food,” “meals,” “help pay for housing,” “help pay for utilities,” “transportation for healthcare,” “help pay for transportation,” “help pay for gas,” “help find housing,” and “temporary shelter.” Search terms for 211 included the following: “emergency food,” “food pantries,” “help paying for food,” “hot meals,” “SNAP/food stamps,” “help paying for electricity,” “help paying for gas,” “help paying for home heating,” “bus passes,” “discounted public transportation,” “free rides,” “emergency shelters,” “help paying for housing,” “homeless,” and “housing vouchers and subsidized housing” (Appendix A).

For each domain, we recorded the total number of resources (n) identified by Aunt Bertha vs 211 for each searched ZIP code. We then mapped the number of resources available in both housing and food domains within each ZIP code to the relative need using ArcGIS 10.1 (Environmental Systems Research Institute, Redlands, CA). Relative need was represented as percent of households in poverty, as identified in the 2018 American Community Survey 5-year estimates (B17017 table) and displayed at the Zip Code Tabulation Areas (ZCTA) level using the 2010 Census ZCTAs. We calculated the mean number of resources across all 51 ZIP codes for each domain. Each resource identified from searches was verified through review of the resource’s website. Resources identified in searches were categorized as correct or incorrect based on whether the resource was related to the domain queried (eg, search under food domain that yielded payment assistance for utility bills was deemed incorrect). Resources were also categorized based on whether eligibility for that resource was demographic-restricted (e g, resource restricted to women, veterans, or children). Given that ZIP codes are in close proximity to one another in this urban area, we did not deem a resource incorrect if it was located outside the specific ZIP code queried.

The number and percentage of resources identified that were correct vs incorrect based on the search domain were recorded and calculated for each ZIP code. To examine how useful resource search results would be in a time-limited ED setting where confirmation around patient eligibility criteria would be challenging, we then calculated the number and percentage of resources correct by domain that were also demographic-restricted. In this way we were able to compare how useful each database would be in an ED setting that may have incomplete information about demographic eligibility (eg, veteran status).

## RESULTS

We identified 30 studies discussing the 211 system, of which 26 were included. Of the four that were excluded upon full-text review, two analyzed the fiscal aspect for the implementation and viability of 211 infrastructure in a state.^21^ One examined data storage variations in 211 centers,^22^ and another described the benefit of using a public library as a 211 center.^23^ The included studies addressed the history and background of the 211 system and role of 211 centers in disaster response and HRSN linkage. We identified 11 articles for review through the NowPow and CommunityRx search. Of these, five were included in final analysis. Six were excluded because they were abstracts only. An extensive search for articles related to Aunt Bertha yielded no results.

### Health Related Social Needs

Reports on the 211 system demonstrated both clinical and research implications. Most people who use 211 are typically a population that is difficult to reach and at high risk of poor health outcomes.^24^,^25^ A Healthcare Navigation Program trial concluded that 211 centers can successfully connect people to resources, particularly in healthcare access.^26^,^27^ The 211 system serves the public by “encouraging healthy behaviors, by raising awareness of preventive services, and by breaking down barriers that prevent access.”^28^ The 211 systems have databases that can inform targeted interventions.^29^ The database combines “anecdotal” details gathered from the personal conversations callers have with 211 staff and “systematic” statistics that are collected for each call in a standard format.^29^

### Disaster Response

A subset of studies focused on the ability of 211 centers to assist communities recovering from disasters. For example, a retrospective study focused on unmet needs in the setting of Hurricanes Katrina and Rita to understand which populations were most vulnerable to resource exhaustion and found that large, metropolitan cities struggled to absorb mass amounts of evacuees even though these areas had extensive community resources.^30^ Additionally, the calls during this time were categorized by the caller’s requested resource to determine the most acutely needed resource.^30^ These authors suggested a similar spatial analysis could be productive to examine the health needs of a given community.^30^

Another study examined 211 centers’ roles during the severe acute respiratory syndrome epidemic and the Great Northeastern Blackout in Toronto, Canada. One lesson extrapolated from these disasters advocated that 211 centers successfully served as a connector for callers and healthcare providers, and thus potentially reduced unnecessary 911 calls and presentations to EDs by connecting callers to services and calming anxieties.^31^ There were no studies identified that assessed the accuracy of the resource databases.

### Results: Resource Database Usability Simulation

**Figure 1:**
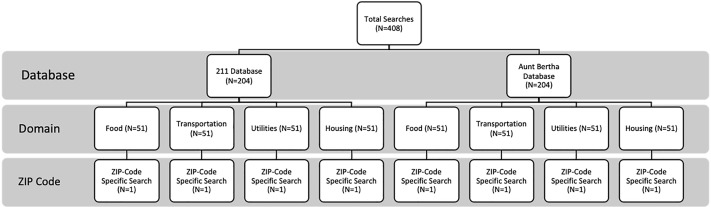
Number of platform searches conducted by database, domain, and ZIP code to identify social needs resources in the emergency department.

We conducted a total of 408 standardized searches across both 211 and Aunt Bertha (Figure 1).

#### Total Number of Resources Identified for Each Domain

Across both databases, the highest average number of resources were identified within the food domain: 211 identified an average of 41 resources per ZIP code for the same simulated search while Aunt Bertha identified an average of 76 resources related to food insecurity per ZIP code. Searches related to transportation needs yielded the fewest average number of resources per ZIP code with 211 identifying 24 resources per ZIP code and Aunt Bertha identifying an average of 22 resources per ZIP code.

#### Geographic Distribution of Resources

**Figure 2:**
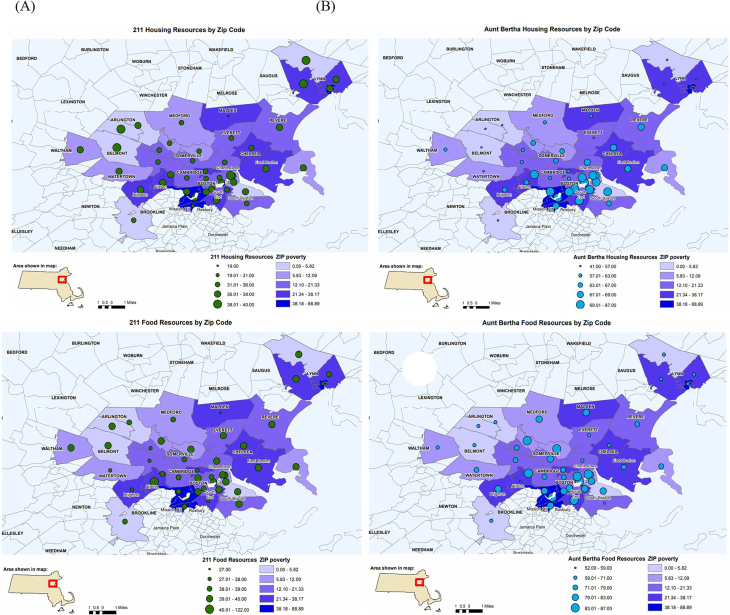
Geographic distribution of resources (n) for each ZIP code relative to poverty index for database housing domain using 211 database (A), housing domain using Aunt Bertha database (B), food domain using 211 database (C), food domain using Aunt Bertha (D). Green circles display 211 resources and blue circles display Aunt Bertha resources. Size of circles represents the number of resources within each ZIP code; larger circles indicate a greater number of resources. Purple scale represents percent of households in poverty within each ZIP code; darker shades of purple indicate greater percentages of poverty.

Across all domains and both databases, the total number of social resources identified varied by ZIP code (Figure 2). The greatest geographical variation in the number of resources per ZIP code was observed under the food domain for both 211 (range = 27–122) and Aunt Bertha (range = 52–108). The least geographic variation in number of resources across ZIP codes occurred within the utilities domain for 211 (range = 38–40) and the transportation domain for Aunt Bertha (range = 19–30) (Figure 2).

#### Accuracy and Applicability Social Resource Referral Databases

Nearly all of the resources identified by both 211 and Aunt Bertha were correct based on the search domain. Averaging across all ZIP codes, both databases had greater than 99% accuracy for each of the four domains assessed. However, a significant proportion of the resources identified by both databases were restricted to a specific demographic. Resources related to housing were most often demographic-restricted; averaging across all ZIP codes and both databases, 54% of resources identified for this domain were found to be demographic-restricted. Conversely, resources related to utilities were the least often demographic-restricted; averaging across both databases, 39% of resources identified for this domain were found to be demographic-restricted.

#### Comparing Across Referral Databases

Averaging across all 51 ZIP codes, 211 identified a fewer number of resources that addressed food insecurity (n = 41) compared to Aunt Bertha (n = 76). However, a higher proportion of the food insecurity resources identified by Aunt Bertha were demographic-restricted relative to 211 (57% vs 32%, respectively). Similarly, under the housing domain, 211 identified only half as many resources on average across ZIP codes relative to Aunt Bertha (n =36 vs n = 68, respectively) but, again, the more resources identified by Aunt Bertha were demographic-restricted (62%) compared to 211 (46%).

Under the transportation domain, 211 identified a greater number of resources compared to Aunt Bertha (n = 24 vs n = 22, respectively); a higher proportion of the resources identified by Aunt Bertha were demographic-restricted relative to 211 (68% vs 17%, respectively). Similarly, 211 identified twice as many resources related to home utilities on average compared to Aunt Bertha (n = 40 vs n = 21); a higher proportion of the resources identified by Aunt Bertha were demographic-restricted relative to 211 (65% vs 13%).

## DISCUSSION

Unmet HRSN are important drivers of disparities in morbidity and mortality during public health emergencies.^2^ Emergency departments need to establish an efficient mechanism to refer patients to community health resources that address their unmet social needs. Geographically indexed database referral systems may represent an effective strategy for quickly and accurately identifying community resources that are within patients’ reach. These resources are available in real time and only require an Internet connection, which is manageable in most EDs. Patients could access the websites or applications by using the hospital’s WiFi and their own device, or the hospital could invest in communal devices located in spaces such as waiting rooms.

Although most public health and screening interventions using geographically indexed community resource referral systems have focused on chronic care needs, these databases have been mobilized during disasters to successfully aid vulnerable populations.^30^,^31^ Additionally, referral systems can be used during disasters to disseminate crucial information to the population and can, in turn, inform EDs of active issues. When recovering from a disaster, collaboration between community resource referral systems and EDs can inform preparation for a similar event in the future.^31^ In addition to connecting patients to resources, data from referral systems can parse out opinions and beliefs held by certain populations. Knowledge of these opinions allows for targeted education efforts designed to increase health efficacy and accessibility for vulnerable populations.

The success of community resource databases in connecting patients with community resources is of importance for EDs attempting to address patients’ HRSN in a time-limited setting with multiple, competing obligations. The ability to access geographically proximate resources for patients in need, and refer them to a free, multilingual hotline for further assistance in accessing resources, has significant potential to improve ED discharge processes, particularly for centers with limited social resources.

An inherent limitation of geographically indexed resource databases is the difficulty ensuring that the referral information is up to date and not specific to certain demographics (eg, veterans). Our standardized searches demonstrated that 39% and 54% of the resources identified by 211 and Aunt Bertha, respectively, were demographic-restricted. In the ED setting, it may not always be feasible for providers to elicit whether a patient meets eligibility criteria for community resources. Therefore, we recommend that ED providers selecting a referral database evaluate the following: ensure that the databases focus on identifying non-demographic-restricted resources and have options to clearly sort by demographic eligibility criteria. Programs should also be clear on the frequency of updates to ensure that patients are successfully referred to operating community resources.

## LIMITATIONS

Our study is subject to a number of limitations. We only simulated use of databases for a single metropolitan area; given geographic variability in availability of community resources, our findings on the relative usability of the two databases we simulated may not be generalizable to other settings, particularly more rural settings. Additionally, relevance of resources to the queried search domain was gleaned from review of the resource’s website and not through direct contact with resource staff, so it is possible that we overestimated the accuracy of some platform search outcomes. Future research should focus on identifying the best strategies for using these resources to address the needs of ED patients. Additionally, future research should also examine the application of these resources to other metropolitan areas and geographic regions.

## CONCLUSION

Health-related social needs are associated with high-frequency utilization of the ED,^4^,^5^ but existing ED systems and resources to address the non-medical, but health-impacting, needs of our patient population are limited. Geographically indexed resources databases have the potential to assist ED providers with identifying appropriate and geographically proximate community resources for patients. Even when applied to the same geographic area, resource databases vary in the total number of resources and the proportion of resources that are not restricted to a particular demographic. Before investing, safety-net organizations should carefully appraise databases to assess their usefulness in meeting their population’s health-related social needs.

## Supplementary Information
