# Supplementary material for: Geographically Indexed Referral Databases to Address Social Needs in the Emergency Department
Source: West J Emerg Med. 2021 Mar 4;22(2):218–24. doi: 10.5811/westjem.2020.11.49250 (PMC7972357; doi:10.5811/westjem.2020.11.49250)
Supplement: Supplementary file 1 [file wjem-22-218-s001.docx]

| Resource Search Terms | | | |
| --- | --- | --- | --- |
| Food | Utilities | Transportation | Housing |
| 211 | | | |
| “Food”   - “emergency food” - “food pantry” - “help pay for food” - “meals” | “Housing”   - “help pay for housing”   - “help pay for utilities” | “Transit"   - “transportation”   - “transportation for healthcare” - “help pay for transportation”   - “help pay for gas” | “Housing”   - “help find housing” - “temporary shelter” |
| Aunt Bertha | | | |
| “Food”   - “Emergency food” - “Food pantries” - “Help paying for food” - “Hot meals” - “SNAP/food stamps” | “Housing”   - “Help paying for electricity” - “Help paying for gas” - “Help paying for home heating” | “Transportation”   - “Bus passes" - “Discounted public transportation” - “Free rides” | “Housing”   - “Emergency shelters” - “Help paying for housing” - “Homeless” - “Housing vouchers and subsidized housing” |

**Appendix A**
